# Annotations

**Published:** 1902-01-18

**Authors:** 


### Annotations.

### Informing the Coroner.

On December 17th a woman fell down and broke her leg. Next day she was conveyed to the Boston Hospital, where on the 29th she suddenly fell back in bed, became speechless and unconscious, and died in about 25 minutes. The medical officer of the hospital came to the conclusion that the deceased had died from the sudden occlusion of a large blood vessel in the brain, and that the fractured leg had not itself caused the death; so he gave a certificate, and all arrangements were made for the funeral. On the circumstances coming to the knowledge of the coroner, however, he stopped the funeral, and held an inquest, in the course of which great stress was laid upon the fact of the coroner not having been informed of the case, and a verdict was given to the effect that the woman died of misadventure, but that "there was great neglect shown by the hospital authorities in not notifying the death of the deceased to the proper quarter." Now, in regard to this case, all one can say is that everything depends upon the wording of the certificate. By courtesy, and for the sake of convenience, no doubt, a system has grown up in many places in accordance with which medical men refuse to give certificates in cases of accident or violence, sending information to the coroner instead. All this, however, is illegal. The doctor is bound by Act of Parliament to give a certificate stating the cause of death "to the best of his knowledge and belief," in every case which dies under his care. Such a certificate ought in cases of accident or violence to disclose the fact, and in such cases it is for the registrar, not the doctor, to inform the coroner. The whole thing is provided for by law. If, then, the certificate in question did not refer to the fact of the accident it did not comply with the law; but if it did, the coroner ought in all fairness to have protected the hospital from such a verdict. The fact is that many coroners have an idea that it is the duty of the doctor to withhold his certificate, and to inform the coroner in all cases of violence, accident or sudden death. As a fact, to withhold the certificate is illegal, while to inform the coroner is no more obligatory upon the doctor than on any other citizen who is aware of the occurrence.

### The Coffee-Drinking Boers.

In an interesting article in this month's *Blackwood* a surgeon who was with the Boers during the siege of Mafeking shows how little they suffered from that disease which has been the bane of our army in South Africa, namely, enteric fever. The force engaged in these operations numbered at first from 6,000 to 7,000. Later on it was considerably smaller. Still, when it is remembered that for several months this force was practically stationary and apparently exposed to the same influences as those which led to such a disastrous prevalence of enteric fever among our troops, the rarity of this disease among the Boers is extremely remarkable, and, unless we may attribute it to the fact that they drank chiefly coffee, which, of course, was boiled, while our troops drank water in whatever state they found it, would seem inexplicable. The writer says: "It would seem to an army medical man almost incredible, but it is nevertheless true that in my laager on the lower Malopo we never had a definite case of enteric." Yet they were encamped on one spot for two months, and then only moved 20 or 30 yards lower down, where they remained for another three or four months. The description given of the filth of the laager, the flies, and the water supply go to show that the same general influences were at work as obtained with us, and yet there was no typhoid. In seeking an explanation one naturally flies to the suggestion that these good Boers being bred and brought up in the midst of filth had all had typhoid, and were thus immune. Unfortunately we have to face the fact that having had the disease by no means gives immunity, and thus we are driven to the suspicion that their freedom from typhoid fever was largely due to their habit of submitting to thirst, putting up with the discomfort until they could obtain their beloved coffee—in other words, boiled water.

### Insanity Among Immigrants.

Protests are not infrequently heard in England concerning the harsh operation of the laws of the United States in reference to the admission of pauper or defective aliens. If, however, we look at the facts of the case we soon find what an immensely important problem this is to a country into which, as is the case in the United States, a constant stream of immigration flows. That there is a something in the climatic and other conditions of the northern States which makes for energy and gives great scope for human activity no one will deny; but the immigrants form the material upon whom these conditions have to exercise their influence; they are the clay which has to be moulded, and on their character depends to a large extent the direction which these activities will take. One can hardly wonder, then, at the anxiety with which the American-born inhabitants of the States regard the undue proportion in which insanity is showing itself among the foreign-born population. It is stated that the number of foreign-born insane persons in American institutions is now considerably more than double what it would be if the proportion so taken care of among the foreign-born population were only the same as that obtaining among those born in the States. In mitigation of the anxiety caused by these facts one has to bear in mind that all neuroses tend to be exaggerated under the influence of such a sudden change as is involved in emigration, and that emigration is often undertaken in consequence of poverty, misfortunes, and troubles of the very kind by which any taint in a nervous system is likely to be displayed. Hence, one may fairly hope that the country which receives these people, when once it has had time to merge their tendencies among those of its own more stable, because longer settled race, will not be so badly off as is suggested by the mental breakdown of so many of its immigrants; still, it is a striking thing to find that 50 per cent. of the inmates of the hospitals for the insane in the New York State are foreign born, while only 25 per cent. of the population of that State is alien. In such facts as these we see enough to explain the anxiety of Americans on the subject, and perhaps to justify them in the somewhat extreme legislative measures which are being proposed with the object of preventing the continuance of such a state of affairs.

